# New directions beyond the boundaries of evidence synthesis

**DOI:** 10.1002/cesm.12005

**Published:** 2023-03-20

**Authors:** Michael Brown, Ella Flemyng

**Affiliations:** ^1^ Department of Emergency Medicine Michigan State University College of Human Medicine Grand Rapids Michigan USA; ^2^ Evidence Production and Methods Directorate Cochrane London UK

Welcome to *Cochrane Evidence Synthesis and Methods*. A new, open‐access journal to facilitate Cochrane's mission of improved health and care decision‐making globally.

Cochrane is an independent and global nonprofit organization committed to producing trusted evidence, advocating for its use, and ensuring it informs health and care decisions. We encourage submissions to *Cochrane Evidence Synthesis and Methods* from Cochrane's community and seek contributions from the evidence synthesis community at large.

We are committed to diversity and inclusivity, and to achieve this we have established an Editorial Board that is geographically dispersed, gender‐balanced and includes people with lived experience, and with a depth of methodological expertise. As the journal develops, we will continue to monitor the representation on our Editorial Board to ensure it reflects the needs of our wider evidence synthesis community.

As Cochrane looks beyond its 30th year [1], *Cochrane Evidence Synthesis and Methods* is a platform that welcomes innovative ideas. This includes how we showcase our commitment to research integrity, including with Open Research Badges [2], embedding consumer involvement within the journal, and improving peer review with a range of different initiatives. We encourage articles that report studies within reviews [3], different types of evidence syntheses that respond to relevant stakeholder questions, for example, gap maps and scoping reviews, and sharing of best practice and case studies to improve efficiencies in evidence synthesis production and ensure our standards are informed by evidence. The editors also encourage accessible language to be used in all submissions, to benefit researchers from across disciplines, nonnative English speakers, and consumers of health.

We are committed to high research integrity and authors will be able to showcase how they adhere to these practices through research integrity indicators. This includes having a clearly stated question with evidence of stakeholder need or research that informed the question to address issues of unnecessary or duplicative research. For the evidence synthesis we receive, we encourage authors to use patient‐important outcomes and/or standardized outcomes, such as those defined by COMET [4]. *Cochrane Evidence Synthesis and Methods* will also follow Cochrane's conflict of interest policy, expect data sharing, when feasible, and adherence to reporting guidelines, as well as encourage statements about consumer involvement in the research. All of this aims to ensure the research we publish is impactful and combats areas of research waste.

Our first published paper is the rapid review on the effect of pharmacological interventions for the treatment of people with post‐COVID‐19 [5], which is an exemplar of the types of rapid reviews we will feature. The authors worked closely with the World Health Organization (WHO), to ensure that the rapid review was relevant, timely, and narrowly focused on therapeutic questions that directly apply to healthcare decisions. Due to timeliness, this rapid review does not include consumers in the author team, however, our Consumer Editor reviewed the plain language summary included in the manuscript.

Other articles in the *Cochrane Evidence Synthesis and Methods* pipeline, deal with health equity, research integrity issues relevant to evidence synthesis, the role of replication in evidence synthesis, and data sharing. We understand that for many authors, timely and fair editorial decisions are a priority, and being flexible and adaptive will be key traits of the journal as we establish ourselves within the community. Authors will also receive proper acknowledgment for their specific contributions to research published in the journal via the CRediT platform [6].

We are keen to support methods training for early and mid‐career researchers. To that end, we have partnered with the Cochrane Methods Support Unit to produce a collection of tutorial articles on common statistical and methodological errors. We aim for these tutorials to be short, relevant, and accessible and are exploring the possibility of publishing short videos and infographics for easy use and digestion of the information.

*Cochrane Evidence Synthesis and Methods* will keep pushing boundaries to improve how we publish and share evidence synthesis and its related research. We look forward to sharing these advancements with you as we set off on our journey with these first publications.

## CONFLICT OF INTEREST STATEMENT

Michael Brown is the Editor of *Cochrane Evidence Synthesis and Methods*. Ella Flemyng is employed by Cochrane and sits on the Editorial Board of *Cochrane Evidence Synthesis and Methods*.
